# Association of remimazolam with delirium and cognitive function in elderly patients undergoing general anesthesia or procedural sedation: a meta-analysis of randomized controlled trials

**DOI:** 10.3389/fmed.2025.1567794

**Published:** 2025-04-28

**Authors:** Yao Wang, Zi-han Gou, Gan-min Wang, Lun-hui Ye, Li Chen, Qian Wang

**Affiliations:** ^1^Department of Anesthesiology, The Thirteenth People’s Hospital of Chongqing, Chongqing, China; ^2^Department of Anesthesiology, The People's Hospital of Kaizhou District Chongqing, Chongqing, China; ^3^Department of Anesthesiology, The Second Affiliated Hospital of Chongqing Medical University, Chongqing, China

**Keywords:** delirium, cognitive function, elderly, remimazolam, postoperative

## Abstract

**Background:**

Remimazolam is an ultra-short-acting benzodiazepine with sedative effects, but its impact on postoperative delirium (POD) and cognitive function in elderly patients remains unclear. This study aimed to compare the incidence of POD and cognitive function between remimazolam and other sedatives in elderly patients undergoing general anesthesia or procedural sedation.

**Methods:**

This study included randomized controlled trials (RCTs) comparing remimazolam with other sedatives in elderly patients undergoing general anesthesia or procedural sedation. A comprehensive search was conducted in Ovid MEDLINE, Embase, the Cochrane Central Register of Controlled Trials, and the China National Knowledge Infrastructure (CNKI) from inception to January 2, 2025, without language restrictions. Data were pooled quantitatively using a random-effects model. The primary outcomes were the incidence of POD and cognitive function.

**Results:**

A total of 1,808 elderly patients from 11 RCTs were included. Compared with other sedatives, remimazolam did not increase the incidence of POD (OR: 0.62, 95% CI [0.23, 1.68], *p* = 0.35, *I*^2^ = 73%), but improve cognitive function, as measured by Mini-Mental State Examination scores, the seventh postoperative day (MD: 0.53, 95% CI [0.16, 0.91], *p* = 0.005, *I*^2^ = 28). Additionally, remimazolam significantly reduced the incidence of hypotension (OR: 0.27, 95% CI [0.21, 0.35], *p* < 0.001, *I*^2^ = 0%) and respiratory depression (OR: 0.35, 95% CI [0.17, 0.69], *p* = 0.003, *I*^2^ = 0%) compared to other sedatives. However, no significant differences were observed between remimazolam and other sedatives for postoperative nausea and vomiting (OR: 1.31, 95% CI [0.91, 1.89], *p* = 0.15, *I*^2^ = 0%) or hypoxemia (OR: 0.69, 95% CI [0.35, 1.34], *p* = 0.28, I^2^ = 0%).

**Conclusion:**

Overall, the use of remimazolam in the elderly population appears to pose fewer risks than other sedatives. It does not increase the incidence of postoperative delirium following general anesthesia or sedation, but it improves postoperative cognitive function and provides more stable hemodynamics. However, further well-designed RCTs with long-term follow-up are needed to establish a standardized medication regimen and optimal dosage tailored to elderly patients.

**Systematic review registration:**

https://www.crd.york.ac.uk/PROSPERO/myprospero, registration number (CRD4202563620).

## 1 Introduction

Postoperative delirium is a common, acute, and transient neurological syndrome that primarily affects elderly individuals (1, 2). It is typically characterized by impaired concentration, altered levels of consciousness, drowsiness, agitation, and aggressive behavior. Elderly patients who experience postoperative delirium are more likely to suffer from perioperative complications, such as accidental removal of wound drainage tubes, wound infections, deep vein thrombosis, and even death (3). These complications not only hinder the recovery process but also increase medical costs and pose significant challenges to the healthcare system in an aging population (4).

With aging, there’s an increase in the nervous system’s responsiveness and alterations in neurotransmitters. The disruption of GABA receptors by benzodiazepines more readily results in consciousness issues among elderly patients (5). Traditional benzodiazepines, known for their extended half-life, may often retain their sedative and cognitive-suppressant properties post-surgery, especially when combined with reduced liver and kidney function in older patients. Additionally, certain benzodiazepines have anticholinergic effects, potentially exacerbating cognitive impairments during therapy (6). Naturally, older patients frequently experience various fundamental health issues, and the interplay among different medications, which can potentially impact cognitive function, is also an aspect that must not be ignored. Based on its pharmacological properties and previous clinical studies (7, 8), individuals tend to consider conventional benzodiazepines as a separate risk factor for postoperative delirium (9), especially in the elderly. Such drugs may result in reduced vigilance, psychomotor impairment, and anterograde amnesia, potentially impairing cognitive function (10). During the perioperative phase, their use should be advised with careful consideration for elderly patients. Recent studies indicate that benzodiazepines are not a primary cause of postoperative delirium (11), and they are still widely used in the perioperative period. Nevertheless, the potential harm of traditional benzodiazepines to elderly patients should not be overlooked, highlighting the urgent need for new, safer sedative drugs with fewer side effects.

Remimazolam is an ultra-short-acting benzodiazepine that induces sedation by binding to central gamma-aminobutyric acid type A receptors (GABA_A). It is increasingly used in various surgical procedures due to its rapid metabolism, short half-life, minimal circulatory effects, and the availability of a specific antagonist (12). Some studies suggest that remimazolam may reduce neuronal inflammation and the incidence of postoperative delirium and cognitive dysfunction (13, 14). However, recent retrospective studies and systematic reviews have found no significant correlation between remimazolam and postoperative delirium in adults (15, 16). In particular, for elderly patients, the association remains unclear due to limited sample sizes and other confounding factors.

To better understand the effects of remimazolam on postoperative delirium and cognitive changes in elderly patients and to enhance its safety in this population, we conducted this comprehensive meta-analysis.

## 2 Methods

### 2.1 Protocol and guidance

This meta-analysis was performed following the established methods recommended by the Cochrane Collaboration. The article adheres to the guidelines of the Preferred Reporting Items for Systematic Reviews and Meta-Analyses (PRISMA). Additionally, the study protocol was prospectively registered on PROSPERO (ID: CRD42025636200).

### 2.2 Criteria for considering studies for this review

Eligible studies were selected based on the following PICOS (participants, interventions, comparators, outcomes, and study design) criteria:

(1) Population: Elderly patients (age ≥65) undergoing general anesthesia or procedural sedation.
(2) Intervention: Use of remimazolam as the primary hypnotic or as an adjunct.
(3) Comparison of intervention: Other hypnotics or sedatives.
(4) Outcome: At least one of the primary outcomes, including the incidence of postoperative delirium or cognitive function. Secondary outcomes included hypotension, postoperative nausea and vomiting, hypoxemia, and respiratory depression.
(5) Study design: Randomized controlled trials.

The studies were excluded based on the following criteria: (1) patients with pre-existing delirium or dementia; (2) patients diagnosed with other neurocognitive or psychiatric disorders; (3) patients with known benzodiazepine allergies; (4) use of other benzodiazepines in perioperative management; or (5) use of remimazolam for the postoperative treatment of delirium or agitation.

### 2.3 Information sources and search strategy

A medical librarian (YW) developed comprehensive search strategies for Ovid MEDLINE, Embase, the Cochrane Central Register of Controlled Trials, and the China National Knowledge Infrastructure (CNKI) from their inception through January 2, 2025. Additionally, the World Health Organization International Clinical Trials Registry Platform was searched for completed but unpublished studies. References from key articles were also screened to identify any additional relevant studies. No restrictions were applied regarding language or publication status. The search terms included “remimazolam,” “hypnotics,” “sedatives,” “elder,” “older,” and “randomized controlled trial,” used individually or in combination. The detailed search strategies are presented in Supplementary Table 1.

### 2.4 Study selection

Two researchers (Z-hG and G-mW) independently assessed the eligibility of studies based on the titles and abstracts retrieved from the electronic search. The full texts of the studies that met the initial criteria were then reviewed independently and in duplicate by both researchers. Disagreements were resolved through consultation with a third researcher (QW).

### 2.5 Data extraction

Two researchers (YW and L-hY) independently extracted data on study characteristics and outcomes from the full-text articles using pretested forms. Disagreements were resolved through consultation with a third researcher (QW).

### 2.6 Assessment of risk of bias and quality of evidence

For each randomized controlled trial (RCT), two researchers (YW and LC) independently assessed the risk of bias using the Cochrane Risk of Bias Tool (RoB 2). The assessment addressed the following domains: random sequence generation, blinding of participants and personnel, allocation concealment, blinding of outcome assessment, selective reporting, incomplete outcome data, and other potential sources of bias. Each domain was categorized as having a low, unclear, or high risk of bias.

The certainty of the evidence regarding the effects of remimazolam in elderly patients undergoing general anesthesia or procedural sedation was evaluated using the Grading of Recommendations, Assessment, Development, and Evaluation (GRADE) approach. This evaluation took into account the risks of bias, inconsistency, imprecision, indirectness, and publication bias in accordance with the detailed GRADE guidelines.

### 2.7 Data synthesis

Statistical analyses were conducted using RevMan (version 5.4, The Cochrane Collaboration) and the meta package in R (version 4.4.2, R Project for Statistical Computing). Pooled treatment effects across studies were estimated using a random-effects model. For dichotomous outcomes, odds ratios (ORs) with 95% confidence intervals (CIs) were computed, while for continuous outcomes, mean differences (MDs) with 95% CIs were reported. Statistical significance was evaluated using two-sided tests, with a *p*-value of < 0.05 deemed significant.

Heterogeneity was assessed visually using forest plots and quantified with *I*^2^ values, categorized as low (0–40%), moderate (40–75%), or high (>75%). Publication bias was not evaluated statistically due to the inclusion of fewer than 10 studies for each outcome.

### 2.8 Subgroup analysis and sensitivity analysis

Subgroup analyses were conducted to identify potential sources of heterogeneity. The prespecified subgroup factors included: (1) type of anesthesia (general anesthesia vs. procedural sedation), (2) American Society of Anesthesiologists (ASA) status, (3) sex distribution (male proportion <50% vs. ≥50%), (4) sample size, and (5) type of surgery (minor, intermediate, and major surgeries). These factors were selected based on their known influence on patients’ responses to anesthesia and recovery, and were considered crucial for evaluating the robustness and reliability of the synthesized results.

Additionally, a leave-one-out sensitivity analysis was performed to assess small-study effects and determine whether any individual study significantly influenced the robustness of the pooled effect size. Furthermore, additional sensitivity analyses for the primary outcomes were conducted using fixed-effect models. Given the heterogeneity of the comparator group, further sensitivity analyses for the primary outcomes were carried out by including only RCTs that compared remimazolam with propofol.

## 3 Results

### 3.1 Study selection and study characteristics

**Figure 1:**
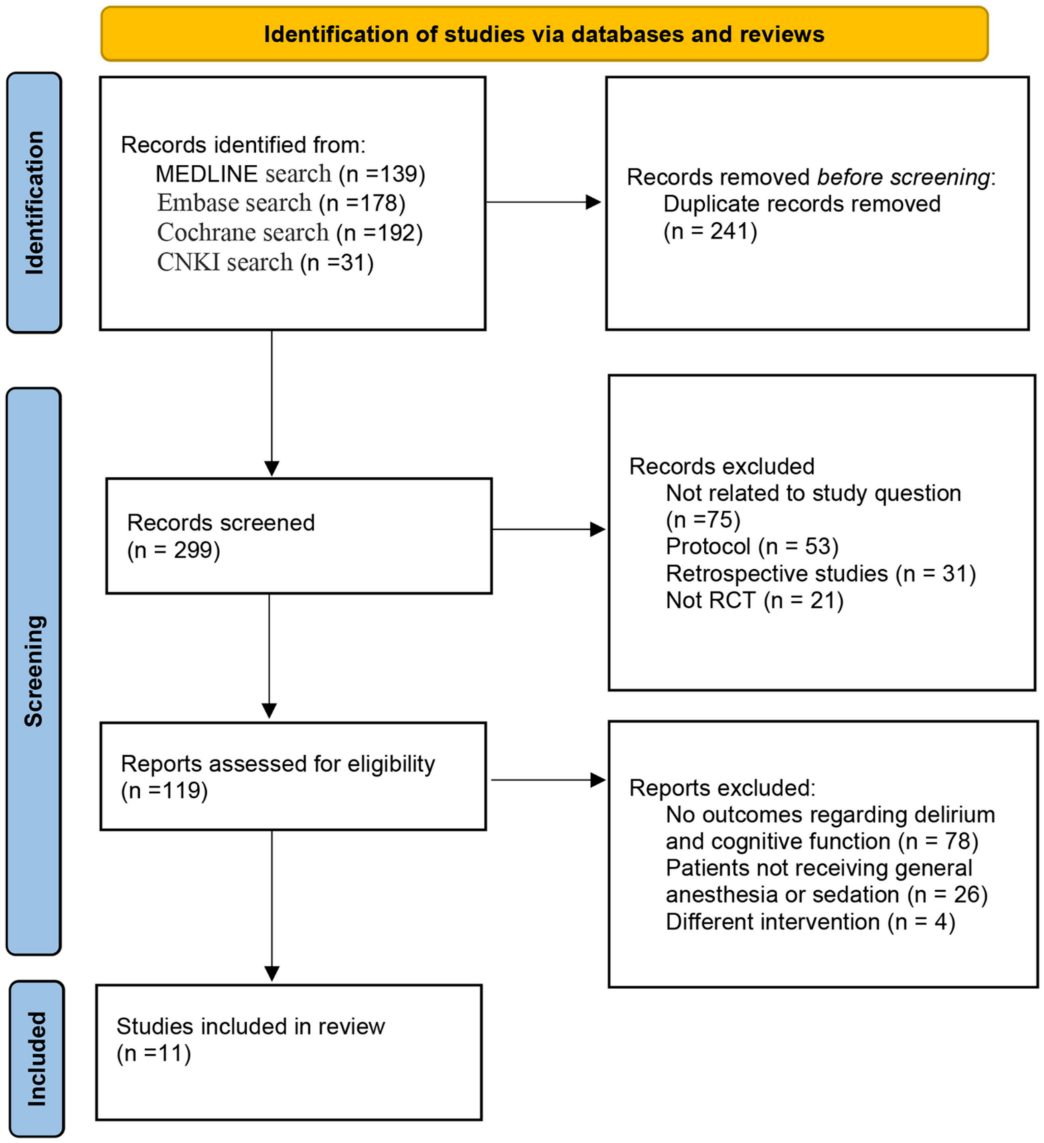
PRISMA flow diagram of study selection.

Figure 1 displays the PRISMA flow diagram for the meta-analysis. A total of 540 potentially eligible publications were initially identified. After removing 241 duplicates, 299 articles were screened based on titles and abstracts, leading to the exclusion of 80 articles. Following a full-text review, 108 studies were further excluded, leaving 11 trials to be included in the final analysis.

The main characteristics of the included studies are summarized in Table 1, which involved a total of 1,808 elderly participants. Sample sizes ranged from 59 to 400 patients. Six studies (17–22) involved patients receiving general anesthesia, while the remaining five studies (23–27) focused on sedation. Propofol was the most commonly used non-benzodiazepine hypnotic in the control group. Postoperative delirium was assessed using the Confusion Assessment Method (CAM) (17, 18, 23), and the Diagnostic and Statistical Manual of Mental Disorders, Fifth Edition (DSM-V) (21). Cognitive function was evaluated using the Mini-Mental State Examination (MMSE) scores in all studies, with Liao et al. (19) additionally employing the Montreal Cognitive Assessment (MoCA). The majority of participants were classified as American Society of Anesthesiologists (ASA) status I or II.

**Table 1:** Baseline characteristics of randomized controlled trials.

References	Number of patients	Intervention	Age (years)	Male (%)	Surgery/procedure	Type of surgery	ASA	Anesthesia
Liu et al. (17)	100	Remimazolam	71.62 ± 5.47	22 (44)	Radical resection of colon cancer	Major	I-III	GA
		Propofol	71.40 ± 5.50	21 (42)				
Duan et al. (34)	106	Remimazolam	77.4 ± 6.1	24 (45)	Hip fracture surgery	Intermediate	II-III	Sedation
		Propofol	75.3 ± 7.7	25 (47)				
Chen et al. (24)	240	Remimazolam+ Sufentanil	71.9 ± 5.0	66 (54)	Gastroscopy	Minor	III-IV	Sedation
		Propofol+ Sufentanil	71.7 ± 5.1	62 (52.5)				
Yang et al. (18)	300	Remimazolam	68 [65 to 71]	61 (41.5)	Orthopedic surgery	Intermediate	I-III	GA
		Propofol	68 [65 to 71]	56 (36.6)				
Liu et al. (25)	216	Remimazolam	67.6 ± 5.7	51 (47.7)	Gastrointestinal endoscopy	Minor	I-III	Sedation
		Propofol	67.5 ± 4.9	51 (46.8)				
Liao et al. (19)	104	Remimazolam+Propofol+Remi	70.12 ± 3.57	21 (61.8)	Laparoscopic radical resection of gastric cancer	Major	NR	GA
		Dex + Propofol+Remi	69.69 ± 2.52	21 (60)				
		Propofol+Remi	71.26 ± 3.58	20 (57.1)				
Kuang et al. (20)	84	Remimazolam	65.4 ± 3.9	19 (45.2)	Thoracoscopic lobectomy	Major	I-II	GA
		Propofol	65.2 ± 4.4	20 (47.6)				
Jeon et al. (21)	122	Remimazolam	70.9 ± 4.3	38 (63.3)	Laparoscopic cholecystectomy and TURBT	Minor	I-III	GA
		Propofol	71.5 ± 4.3	40 (64.5)				
Zhang et al. (22)	59	Remimazolam	74.31 ± 10.6	11 (36.6)	Hip replacement	Major	II-III	GA
		Propofol	75.04 ± 9.98	12 (41.3)				
Lu at al. (26)	400	Remimazolam	70.6 ± 4.7	78 (39.0)	Gastrointestinal endoscopy	Minor	I-II	Sedation
		Propofol	70.1 ± 4.5	83 (46.5)				
Guo et al. (27)	77	Remimazolam	70.4 ± 3.9	25 (64.1)	Gastrointestinal endoscopy	Minor	I-II	Sedation
		Propofol	69.1 ± 4.0	22 (57.9)				

Dex, Demedetomidine; Remi, remifentanil; ASA, American Society of Anesthesiologists; GA, general anesthesia; TURBT, transurethral resection of bladder tumor.

### 3.2 Risk of bias and quality of evidence

**Figure 2:**
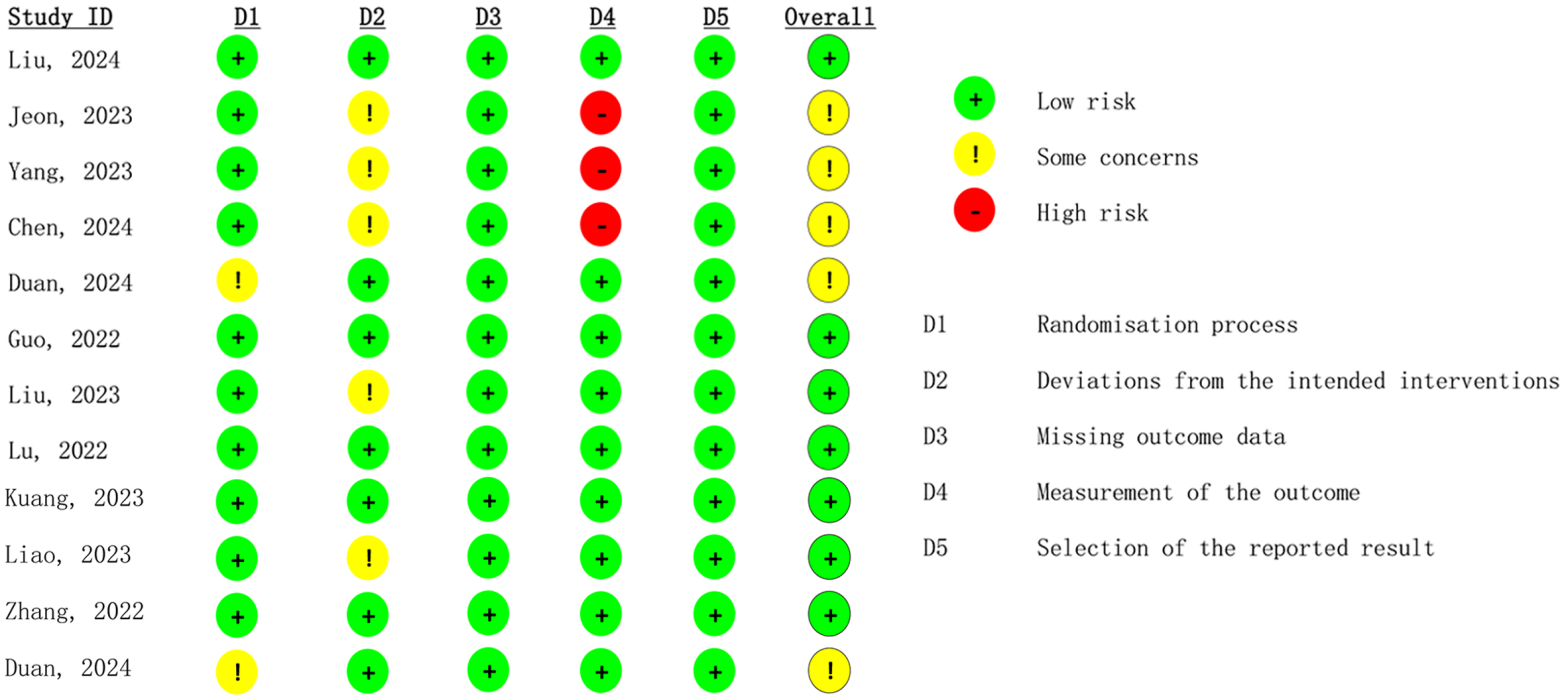
Risk-of-bias results of include studies.

The risk-of-bias assessments are presented in Figure 2. Most domains in the included studies showed a low risk of bias; however, attention is warranted for deviations from intended interventions and outcome measurements, as several studies exhibited a high risk of bias.

The GRADE summary findings for all outcomes are presented in Supplementary Table 2. The quality of evidence for postoperative delirium and cognitive function on postoperative day 7 was rated as moderate. Publication bias was not assessed due to the limited number of studies (<10) included for each outcome.

### 3.3 Postoperative delirium

**Figure 3:**
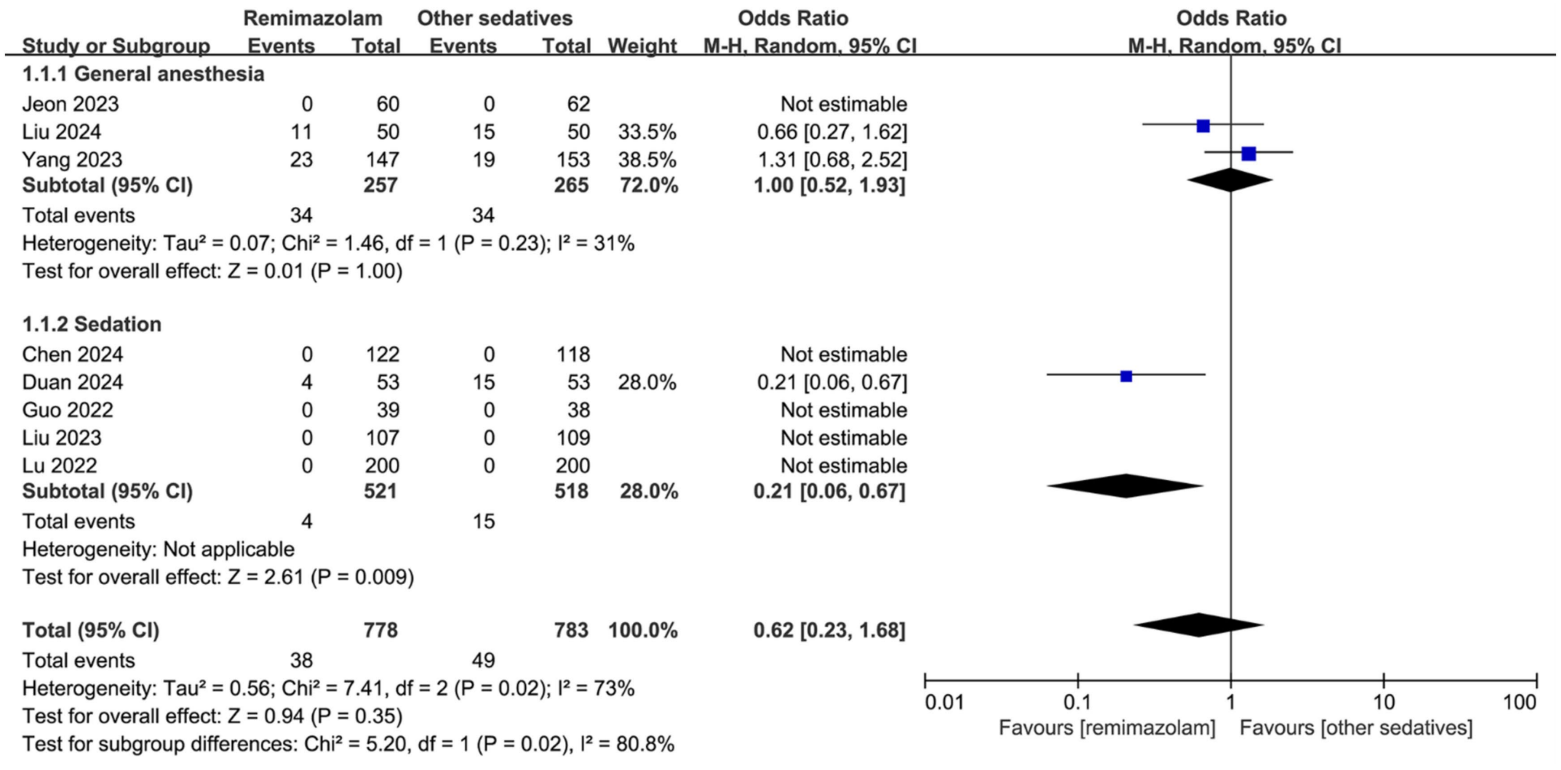
Forest plot for the incidence of postoperative delirium. CI, confidence interval.

Eight trials involving 1,561 patients reported data on postoperative delirium. The time window and frequency of delirium assessments in these trials are detailed in Supplementary Table 3. The overall incidence of postoperative delirium was 4.9% in the remimazolam group, compared to 6.3% in the other sedatives group. The pooled effect size showed that remimazolam administration did not significantly increase the incidence of postoperative delirium (OR: 0.62, 95% CI [0.23, 1.68], *p* = 0.35, *I*^2^ = 73%; Figure 3).

In a subgroup analysis of three studies involving elderly patients undergoing general anesthesia, there was no significant difference in the incidence of delirium between remimazolam and other sedatives (OR: 1.00, 95% CI [0.52, 1.93], *p* = 0.23, *I*^2^ = 31%; Figure 3). However, remimazolam was significantly associated with a lower risk of postoperative delirium compared to other sedatives following procedural sedation (OR: 0.21, 95% CI [0.06, 0.67], *p* = 0.02, *I*^2^ = 73%; Figure 3). The interaction between anesthesia type (general anesthesia vs. procedural sedation) and remimazolam on postoperative delirium was statistically significant (*p* for interaction = 0.02).

Other subgroup analyses of postoperative delirium revealed no significant interactions with variables such as ASA status (I-III vs. III-IV) (The interaction term was not applicable), sex (The interaction term was not applicable), sample size (<200 vs. ≥200) (*p* for interaction = 0.07), or type of surgery (*p* for interaction = 0.87); (Supplementary Figure 1).

A sensitivity analysis demonstrated that the pooled OR remained consistent after sequentially omitting individual studies, confirming the robustness of the findings (Supplementary Figure 2). Furthermore, the incidence of delirium did not differ significantly between remimazolam and other sedatives, as assessed using fixed-effect models (OR: 0.75, 95% CI [0.47, 1.20], *p* = 0.23, *I*^2^ = 73%). Similarly, no significant difference was observed in the incidence of delirium between remimazolam and propofol (OR: 0.62, 95% CI [0.23, 1.68], *p* = 0.35, *I*^2^ = 73%).

### 3.4 Postoperative cognitive function

**Figure 4:**
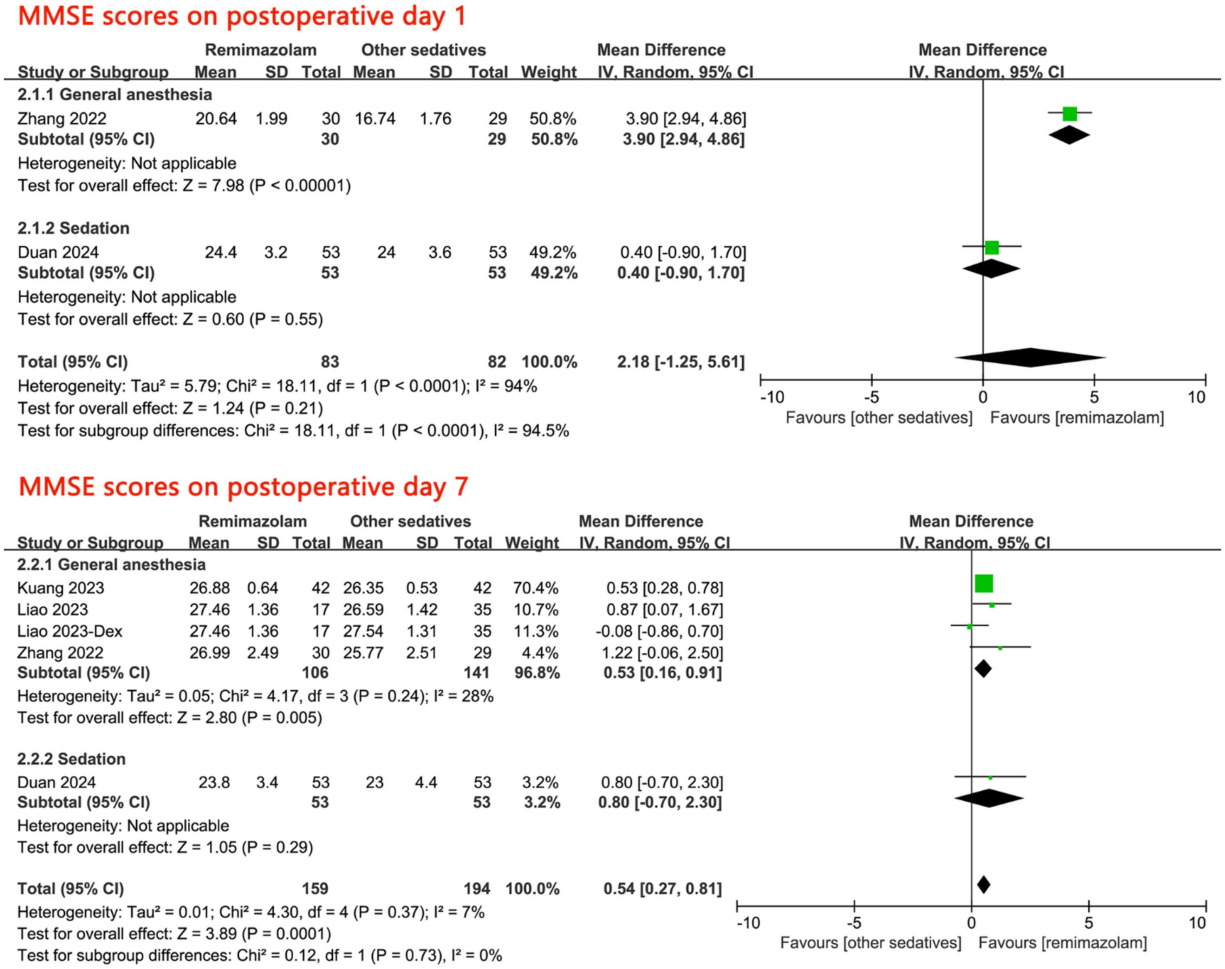
Forest plot for postoperative cognitive function. MMSE, Mini-Mental State Examination; Dex, Demedetomidine; CI, confidence interval.

Four trials involving 353 patients reported data on postoperative cognitive function. Overall, there was no significant difference in postoperative cognitive function, as measured by MMSE scores on the first postoperative day, between remimazolam and other sedatives (MD: 2.18, 95% CI [−1.25, 5.61], *p* = 0.21, *I*^2^ = 94%; Figure 4). However, among patients undergoing general anesthesia, remimazolam demonstrated a significant advantage over other sedatives (MD: 3.90, 95% CI [2.94, 4.86], *p* < 0.001, Figure 4).

By the seventh postoperative day, cognitive function was significantly higher in the remimazolam group compared to the other sedatives group. Specifically, for patients receiving general anesthesia, remimazolam maintained its superiority (MD: 0.53, 95% CI [0.16, 0.91], *p* = 0.005, *I*^2^ = 28%; Figure 4). However, this association was not observed in patients receiving procedural sedation (MD: 0.80, 95% CI [−0.70, 2.30]; Figure 4).

Subgroup analyses of cognitive function on the seventh postoperative day revealed no significant interactions with variables such as type of anesthesia (*p* for interaction = 0.73), ASA status (I-II vs. II-III) (*p* for interaction = 0.32), sex (*p* for interaction = 0.73), sample size (<100 vs. ≥100) (*p* for interaction = 0.75), or type of surgery (*p* for interaction = 0.73); (Supplementary Figure 3).

A leave-one-out sensitivity analysis of cognitive function on the seventh postoperative day showed that remimazolam did not demonstrate superiority over other sedatives after excluding the trial by Kuang et al. However, the pooled MD remained consistent when each of the other trials was sequentially omitted, supporting the robustness of the results (Supplementary Figure 4). Additionally, cognitive function remained significantly higher in the remimazolam group compared to the other sedatives group on the seventh postoperative day, as assessed using fixed-effect models (MD: 0.53, 95% CI [0.31, 0.76], *p* < 0.001, *I*^2^ = 7%). Similarly, cognitive function was significantly higher in the remimazolam group compared to the propofol group on the seventh postoperative day (MD: 0.56, 95% CI [0.32, 0.81], *p* < 0.001, *I*^2^ = 0%).

### 3.5 Secondary outcomes

**Figure 5:**
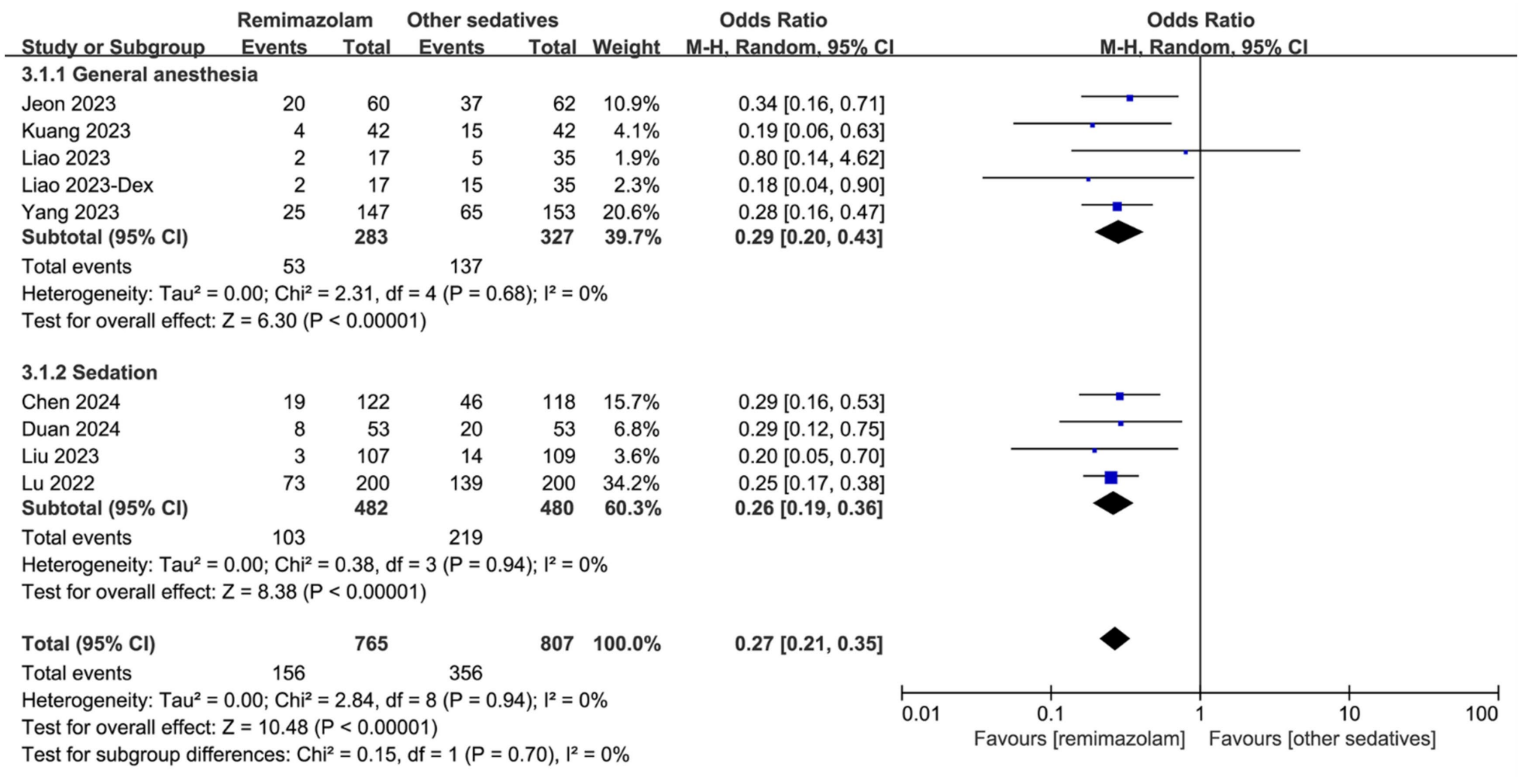
Forest plot for the incidence of hypotension during surgery. Dex, Demedetomidine; CI, confidence interval.

The incidence of hypotension during surgery is shown in Figure 5. Overall, remimazolam significantly reduced the incidence of hypotension compared to other sedatives (OR: 0.27, 95% CI [0.21, 0.35], *p* < 0.001, *I*^2^ = 0%; Figure 5). This effect was consistent in both the general anesthesia subgroup (OR: 0.29, 95% CI [0.20, 0.43], *p* < 0.001, *I*^2^ = 0%; Figure 5) and the sedation subgroup (OR: 0.26, 95% CI [0.19, 0.36], *p* < 0.001, *I*^2^ = 0%; Figure 5).

Other outcomes, including postoperative nausea and vomiting, hypoxemia, and respiratory depression, are presented in Supplementary Figure 5. Specifically, the definitions of respiratory depression used in the included RCTs were detailed in Supplementary Table 4. No significant differences were found between remimazolam and other sedatives for postoperative nausea and vomiting (OR: 1.31, 95% CI [0.91, 1.89], *p* = 0.15, *I*^2^ = 0%) or hypoxemia (OR: 0.69, 95% CI [0.35, 1.34], *p* = 0.28, *I*^2^ = 0%). However, remimazolam was associated with a significantly lower incidence of respiratory depression compared to other sedatives (OR: 0.35, 95% CI [0.17, 0.69], *p* = 0.003, *I*^2^ = 0%).

## 4 Discussion

Our meta-analysis included 11 randomized controlled trials (RCTs), and the results indicated that while remimazolam does not significantly reduce the incidence of postoperative delirium in this population, it notably improves cognitive function by the seventh postoperative day. Additionally, remimazolam was associated with a reduced risk of intraoperative hypotension and respiratory complications during surgery. These benefits seem to contribute to maintaining a steadier blood oxygen flow to the brain throughout the perioperative phase, which may adjust the prolonged cognitive condition of older patients. These advantages collectively highlight its potential as a safer anesthetic.

Relative to other populations, elderly patients tend to suffer more from delirium and consciousness issues post-surgery, often due to pathological and physiological alterations like reduced central nervous system activity. However, earlier research focusing on remimazolam and POD lacks a comprehensive examination and discussion of this particular group. Given the increasing use of remimazolam in medical practice in recent years and the acceleration of population aging, it is necessary to closely monitor the potential impact of remimazolam on elderly patients. Consequently, our research focused on elderly patients, aiming to bridge the research gap by assessing remimazolam’s impact on cognitive function and the occurrence of delirium post-surgery.

The American Geriatrics Society has highlighted the increased risk of delirium in older adults associated with anticholinergic drugs, extended-release benzodiazepines, and opioids like ketamine, advising caution in their use (28, 29). However, the widespread use of benzodiazepines and opioids in surgical settings continues across various patient groups, largely due to their unique pharmacological benefits and irreplaceability, particularly for sedation and the treatment of anxiety in elderly patients. In our study, the use of remimazolam in elderly patients did not result in statistically significant differences in the incidence of delirium. However, there was moderate heterogeneity (73%) in the incidence of delirium. We observed a statistically significant interaction between anesthesia type (general anesthesia vs. procedural sedation) and remimazolam on postoperative delirium (P for interaction = 0.02). But the interaction suggests that the effect of remimazolam on the incidence of postoperative delirium may vary depending on the type of anesthesia used. However, the finding that remimazolam reduces the incidence of postoperative delirium in procedural sedation is based on a single small study (*n* = 106), and further research is needed to confirm these results.

Although some studies suggested that remimazolam may alleviate delirium symptoms in elderly patients following surgery (23, 30), our analysis and other scientific research (15, 16, 31) conducted in adults consistently indicate that remimazolam does not offer a significant advantage in reducing postoperative delirium. Subgroup analysis revealed no intrinsic link between the type of anesthesia, gender, and postoperative cognitive function. However, elderly patients who underwent general anesthesia had a significantly higher likelihood of developing postoperative delirium compared to those who received sedation. This implies that the variety and total amount of anesthesia medications play crucial roles that must not be overlooked when developing POD in elderly patients. Up until now, the precise impact of remimazolam on postoperative delirium and cognitive function in elderly patients has remained largely undefined. Despite our discovery that remimazolam enhances cognitive abilities on the seventh day post-surgery, the lack of long-term consciousness-state monitoring data necessitates further extensive research to understand its precise effects on the consciousness state in older patients.

Remimazolam, an innovative ultra-short-acting benzodiazepine, does not significantly increase the risk of POD in elderly patients compared to sedatives like propofol. We believe this may be attributed to several key factors outlined below: first, remimazolam is rapidly metabolized by tissue esterases into inactive carboxylic acid metabolites, thus preventing the accumulation of active metabolites. And its elimination half-life after a single injection is less than 1 h, meaning that even elderly patients with liver or kidney dysfunction can minimize its persistent depressive effects on the nervous system (32). Second, its observed properties in multiple studies, including promoting the recovery of cellular immune function (33), antioxidant (34), and anti-inflammatory (35) properties, as seen in fundamental experimental studies, appear to offer neuroprotective benefits. Furthermore, the reduced regulation of blood circulation in the brain in the elderly significantly contributes to the development of POD. Compared to other sedatives, remimazolam ensures steadier blood flow to the brain during surgical procedures, enhancing neuroprotection. Moreover, the safety of remimazolam is further enhanced by flumazenil, a specific antagonist that rapidly reverses its neuroinhibitory effects (36). These make remimazolam a potentially safer option for the elderly population.

Our analysis indicates that, compared to other anesthetics, remimazolam provides more stable organ blood flow during surgery in elderly patients and reduces the risk of respiratory depression. These factors may play a critical role in preserving cognitive function after surgery (16, 31). While no statistically significant differences were observed between remimazolam and non-benzodiazepine drugs in cognitive function on the first postoperative day, patients treated with remimazolam demonstrated significantly better cognitive function by day 7. The results indicated that remimazolam might be preferred as a sedative in clinical settings for older patients, particularly for those at elevated risk of POD, including individuals susceptible to respiratory depression, obesity, frailty, hemodynamic instability, prolonged sedative use, or concurrent neurological conditions. Patients in this category often favor sedatives that barely affect breathing or blood flow.

Our systematic review and meta-analysis has several limitations. First, the limited sample size (11 RCTs) may affect the reliability and robustness of the findings. Second, most studies were conducted in China, limiting generalizability due to variations in medical practices, regions, and ethnicities. Broader research across diverse populations is needed. Third, the lack of standardized definitions and tools for assessing postoperative delirium may impact the accuracy of the results. For example, in the postoperative cognitive function assessment, the MMSE scale is mostly used, but it has the disadvantage of low sensitivity to mild cognitive impairment. Future research might explore employing evaluation instruments that are more attuned to the needs of older patients, like the Montreal Cognitive Assessment. Fourth, the variation in the time window and frequency of postoperative delirium assessments across the included trials may influence the evaluation of remimazolam’s impact on postoperative delirium. Fifth, the definitions used in the included RCTs showed slight variations, which may potentially affect the evaluation of remimazolam’s impact on respiratory depression. Additionally, the limited sample size highlights the need for further research on this topic. Lastly, variations in remimazolam regimens, including dosage and duration, contribute to heterogeneity. These factors warrant caution when interpreting the results.

## 5 Conclusion

Overall, the use of remimazolam in the elderly population appears to pose fewer risks than other sedatives. It does not increase the incidence of postoperative delirium following general anesthesia or sedation, but it improves postoperative cognitive function and provides more stable hemodynamics. This could render it a safer medication option for fragile, severely sick older patients. Nonetheless, due to the limited existing experience and studies on remimazolam’s application in older adults, there’s a need for more meticulously planned randomized controlled trials (RCTs) and extended monitoring to formulate uniform medication protocols and ideal dosages for these patients.

## Data Availability

The original contributions presented in the study are included in the article/Supplementary material, further inquiries can be directed to the corresponding author.
