# Supplementary material for: Association of remimazolam with delirium and cognitive function in elderly patients undergoing general anesthesia or procedural sedation: a meta-analysis of randomized controlled trials
Source: Front Med (Lausanne). 2025 Apr 28;12:1567794. doi: 10.3389/fmed.2025.1567794 (PMC12066618; doi:10.3389/fmed.2025.1567794)
Supplement: Supplementary file 1 [file Data_sheet_1.docx]

# *Supplementary Material*

**Supplementary Table 1** **Ovid MEDLINE search strategy used to identify original randomized controlled trials comparing remimazolam with other sedatives in patients undergoing general anesthesia or procedural sedation. (The searches conducted on the Cochrane Central Register of Controlled Trials and EMBASE included slight variations)**

| **Search** | **Query** | **Items found** |
| --- | --- | --- |
| #1 | Remimazolam | 812 |
| #2 | ONO 2745 | 812 |
| #3 | ONO2745 | 812 |
| #4 | CNS7056 | 6 |
| #5 | #1 OR #2 OR #3 OR #4 | 813 |
| #6 | Propofol | 27,506 |
| #7 | Ketamine | 26,931 |
| #8 | Etomidate | 3,227 |
| #9 | Dexmedetomidine [Mesh] OR dexmedetomidine | 10,284 |
| #10 | Sevoflurane | 11,862 |
| #11 | Isoflurane | 16,171 |
| #12 | Methyl Ethers OR Methyl Ether | 42,540 |
| #13 | Hypnotics and Sedatives OR Hypnotics and Sedatives [Mesh] | 135,810 |
| #14 | Ketamine [Mesh] | 16,072 |
| #15 | Esketamine [Supplementary Concept] | 683 |
| #16 | #6 OR #7 OR #8 OR #9 OR #10 OR #11 OR #12 OR #13 OR #14 OR #15 | 212,178 |
| #17 | Anesthesia, general | 124,309 |
| #18 | Anesthesia [Mesh] | 211,813 |
| #19 | Anaesthesia | 452,367 |
| #20 | Anesthesia and Analgesia | 284,890 |
| #21 | Sedation | 59,823 |
| #22 | Sedative | 149,075 |
| #23 | #17 OR #18 OR #19 OR #20 OR #21 OR #22 | 632,945 |
| #24 | Older OR Aged OR elderly | 6,543,518 |
| #25 | randomized controlled trial[pt] OR controlled clinical trial[pt] OR clinical trials as topic[mesh:noexp] OR trial[ti] OR random*[tiab] OR placebo*[tiab] | 2,077,900 |
| #6 | #5 AND #16 AND #23 AND #24 AND #25 | 139 |

**Supplementary Table 2.** **Summary of findings and strength of evidence in studies comparing remimazolam vs. other sedatives in adults undergoing general anesthesia or procedural sedation**

| **Outcome** | **No. of patients**  **(Studies)** | **Relative effect (95% CI)** | **I^2^** | **Absolute effect estimates** | **Quality** |
| --- | --- | --- | --- | --- | --- |
| Postoperative delirium | 1561 (8) | OR 0.62  (0.12 to 1.68) | 73% | 23 fewer per 1000 (from 47 fewer to 38 more) | Moderate^1^ |
| Postoperative cognitive function on day1 | 165 (2) | MD 2.18  (-1.25 to 5.61) | 94% | MD 2.18 higher (1.25 lower to 5.61 higher) | Low^1,2^ |
| Postoperative cognitive function on day7 | 353 (4) | MD 0.54  (0.27 to 0.81) | 7% | MD 0.54 higher (0.27 to 0.81 higher) | Moderate^1^ |
| Hypotension | 1572 (8) | OR 0.27  (0.21 to 0.35) | 0% | 265 fewer per 1000 (from 225 fewer to 299 fewer) | Moderate^1^ |
| Postoperative nausea and vomiting | 1602 (9) | OR 1.31  (0.91 to 1.89) | 0% | 22 more per 1000 (from 7 fewer to 61 more) | Moderate^1^ |
| Hypoxemia | 965 (6) | OR 0.69  (0.35 to 1.34) | 0% | 14 fewer per 1000 (from 29 fewer to 15 more) | Moderate^1^ |
| Respiratory depression | 921 (6) | OR 0.35  (0.17 to 0.69) | 0% | 44 fewer per 1000 (from 20 fewer to 57 fewer) | High |

CI: Confidence interval; OR: Odds ratio; MD: Mean difference; NA: not applicable

^1^ risk of bias

^2^ inconsistency

**Supplementary Table 3. The time window and frequency of assessment for postoperative delirium in the included RCTs**

| **Author, year** | **Time window** | **Frequency** |
| --- | --- | --- |
| Liu,  2024 | within 7 days postoperatively | Twice daily |
| Duan,  2024 | within 7 days postoperatively | Once daily |
| Chen,  2024 | NR | NR |
| Yang,  2023 | within 3 days postoperatively | Twice daily |
| Liu,  2023 | NR | NR |
| Jeon,  2023 | within the first day postoperatively | NR |
| Lu,  2022 | NR | NR |
| Guo,  2022 | NR | NR |

NR: not reported.

**Supplementary Table 4. The definitions of respiratory depression in the included RCTs**

| **Author, year** | **The definitions of respiratory depression** |
| --- | --- |
| Chen, 2024 | Respiratory rate (RR) ≤ 8 times/min or peripheral oxygen saturation (SpO2) < 90 |
| Guo, 2022 | Respiratory rate < 8 times per min and/or blood oxygen saturation < 90% |
| Liao, 2023 | NR |
| Liu, 2024 | Respiratory rate of ≤10 times per minute |
| Lu, 2022 | Respiratory rate < 8 per minute and/or SpO2 < 90% |

NR: not reported.


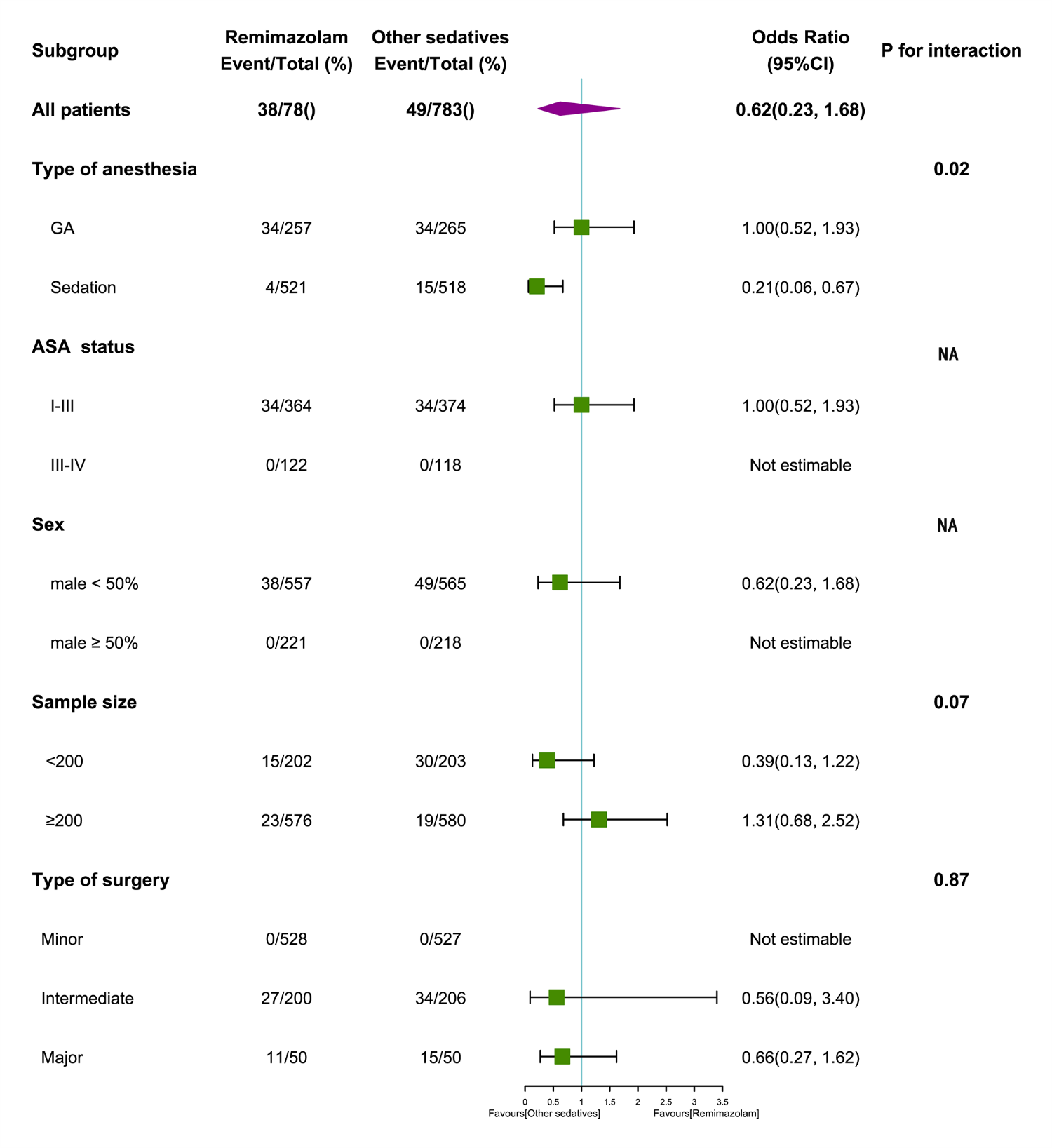


**Supplementary Figure 1. Subgroup analysis for** **postoperative delirium**

GA: general anesthesia; ASA: American Society of Anesthesiologists; CI: confidence interval


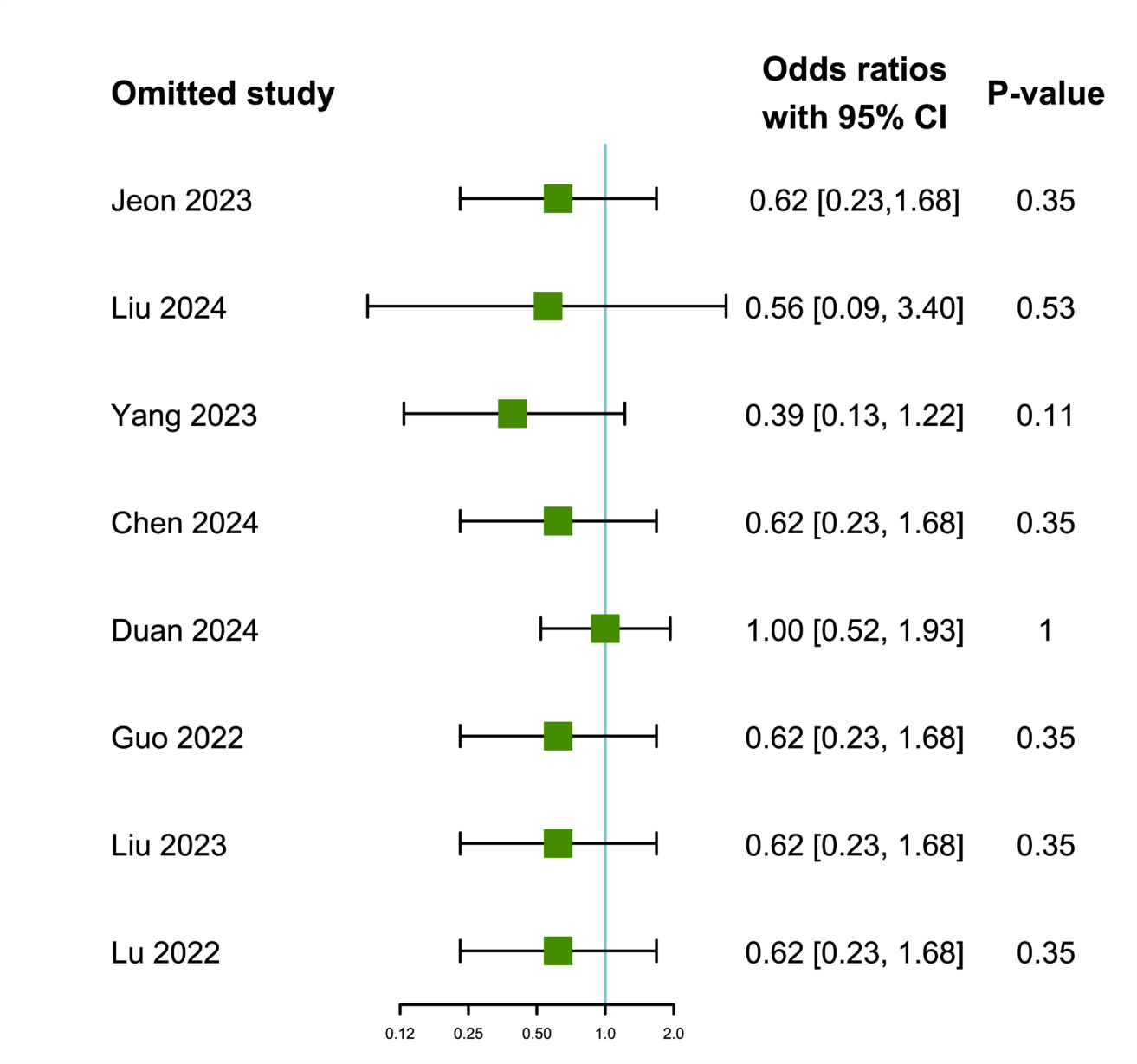


**Supplementary Figure 2. Forest plot for the sensitivity analysis of the incidence of postoperative delirium**

CI: confidence interval.


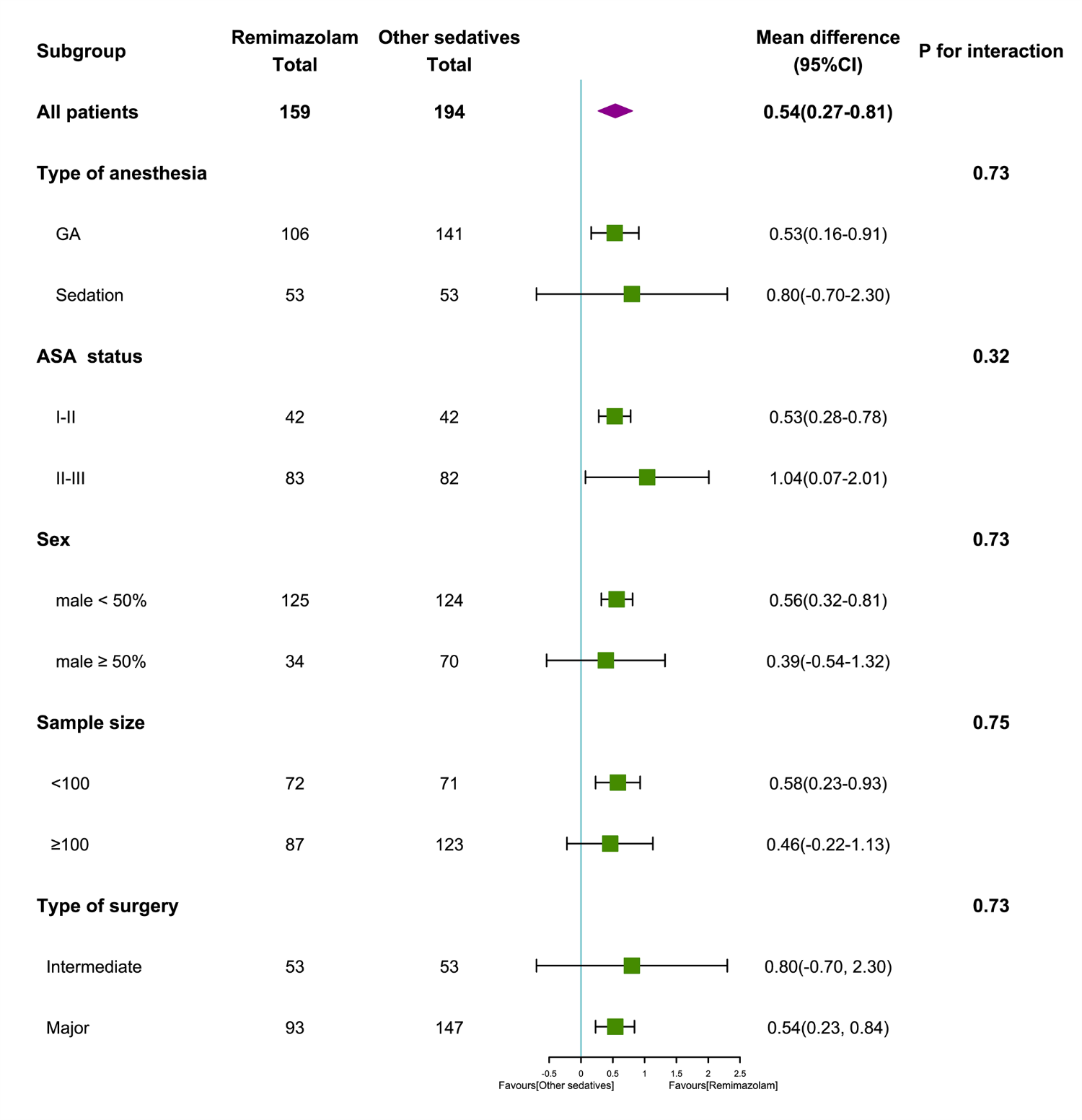


**Supplementary Figure 3. Subgroup analysis for** **postoperative cognitive function on postoperative day 7**

GA: general anesthesia; ASA: American Society of Anesthesiologists; CI: confidence interval


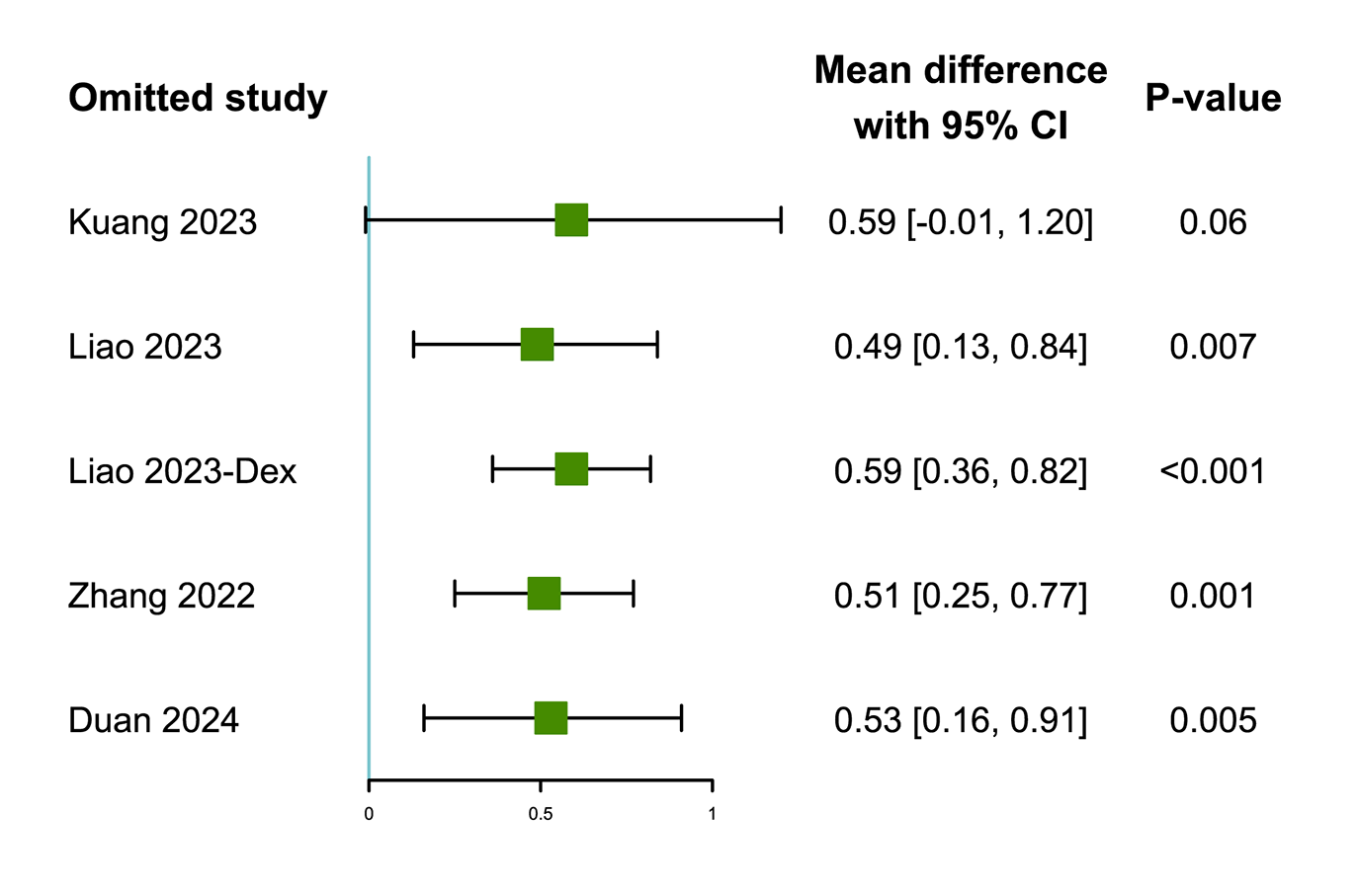


**Supplementary Figure 4. Forest plot for the sensitivity analysis of postoperative cognitive function on postoperative day 7**

Dex: Demedetomidine; CI: confidence interval.


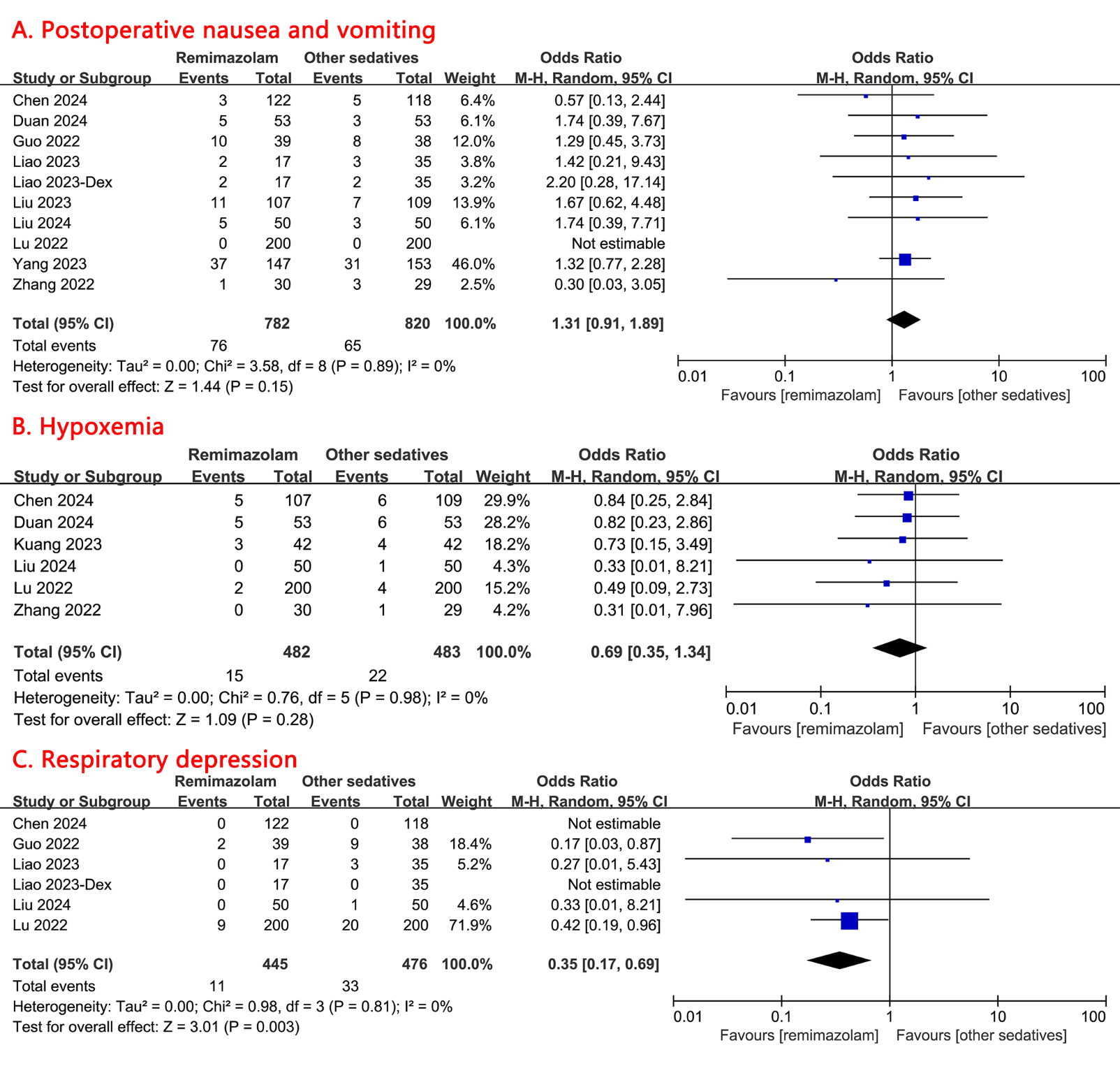


**Supplementary Figure 5. Forest plot for the incidence of (A) postoperative nausea and vomiting, (B) hypoxemia, and (C) respiratory depression**

Dex: Demedetomidine; CI: confidence interval.
